# Elevated Prostaglandin E_2_ Synthesis Is Associated with Clinical and Radiological Disease Severity in Cystic Fibrosis

**DOI:** 10.3390/jcm13072050

**Published:** 2024-04-02

**Authors:** Silvia Gartner, Jordi Roca-Ferrer, Paula Fernandez-Alvarez, Isabel Lima, Sandra Rovira-Amigo, Elena García-Arumi, Eduardo F. Tizzano, César Picado

**Affiliations:** 1 Unidad de Neumología Pediátrica y Fibrosis Quística, Hospital Vall d’Hebrón, Universitat Autònoma de Barcelona, 08035 Barcelona, Spain; silvia.gartner@vallhebron.cat (S.G.); ilima.hcme.ste@gencat.cat (I.L.); sandra.rovira@vallhebron.cat (S.R.-A.); 2 Hospital Clinic, Universitat de Barcelona, 08036 Barcelona, Spain; jrocaf@recerca.clinic.cat; 3 Institut d’Investigacions Biomèdiques August Pi i Sunyer (IDIBAPS), 08036 Barcelona, Spain; 4 Centro de Investigaciones en Red de Enfermedades Respiratorias (CIBERES), 28029 Madrid, Spain; 5 Área de Genética Clínica y Molecular, Hospital Vall d’Hebrón, Universitat Autònoma de Barcelona, 08035 Barcelona, Spain; paula.fernandez@vallhebron.cat (P.F.-A.); elena.garcia@vallhebron.cat (E.G.-A.); eduardo.tizzano@vallhebron.cat (E.F.T.); 6 Medicina Genética, Vall d’Hebrón Institut de Recerca VHIR, 08035 Barcelona, Spain

**Keywords:** air trapping, bronchiectasis, CF transmembrane conductance regulator gene, cystic fibrosis, polymorphisms, prostaglandin E_2_, prostaglandin D_2_, tetranor-PGEM, tetranor-PGDM

## Abstract

**Background:** Previous studies found high but very variable levels of tetranor-PGEM and PGDM (urine metabolites of prostaglandin (PG) E_2_ and PGD_2_, respectively) in persons with cystic fibrosis (pwCF). This study aims to assess the role of cyclooxygenase COX-1 and COX-2 genetic polymorphisms in PG production and of PG metabolites as potential markers of symptoms’ severity and imaging findings. **Methods:** A total of 30 healthy subjects and 103 pwCF were included in this study. Clinical and radiological CF severity was evaluated using clinical scoring methods and chest computed tomography (CT), respectively. Urine metabolites were measured using liquid chromatography/tandem mass spectrometry. Variants in the *COX-1* gene (PTGS1 639 C>A, PTGS1 762+14delA and *COX-2* gene: PTGS2-899G>C (-765G>C) and PTGS2 (8473T>C) were also analyzed. **Results:** PGE-M and PGD-M urine concentrations were significantly higher in pwCF than in controls. There were also statistically significant differences between clinically mild and moderate disease and severe disease. Patients with bronchiectasis and/or air trapping had higher PGE-M levels than patients without these complications. The four polymorphisms did not associate with clinical severity, air trapping, bronchiectasis, or urinary PG levels. **Conclusions:** These results suggest that urinary PG level testing can be used as a biomarker of CF severity. COX genetic polymorphisms are not involved in the variability of PG production.

## 1. Introduction

Cystic fibrosis (CF) is caused by mutations within the CF transmembrane conductance regulator (*CFTR*) gene leading to defective epithelial chloride transport in many organs. More than 2114 variants in the *CFTR* gene have been identified [1]. Various variants can be grouped into different classes based on their known or predicted molecular mechanisms of dysfunction and the functional consequences for the CFTR protein [2].

Class I: pathogenic variants in this category are associated with a lack of biosynthesis or defective biosynthesis, resulting in no CFTR protein. Class II: these gene variants fail to properly process the protein to a mature form and fail to transport the protein to the apical membrane. Class III: variants of this class affect the regulation of CFTR function by preventing ATP binding-induced nucleotide-binding dimerization resulting in the impaired gating of the CFTR channel. Class IV: variants affect the chloride conductance resulting in a normal amount of CFTR with some residual function at the apical membrane. Class V: variants associated with the reduced biosynthesis of a fully active CFTR protein. Class VI: variants destabilize the CFTR protein at the cell surface and produce a high turnover of CFTR. Class VII: large deletions in the CFTR gene resulting in impaired mRNA production.

A graduated risk of developing pancreatic insufficiency and pancreatitis, according to genotype severity, has been reported in various studies [3,4]. Class I, II, and III include pathogenic variants usually associated with pancreatic insufficiency and, thus, are considered severe variants, while class IV, V, and VI variants retain residual function and are usually associated with normal or slightly altered pancreatic function, being classified as mild [3,4]. Numerous studies assessing the impact of CFTR variants on the severity and progression of lung disease have presented discrepant results, some showing correlations between CFTR genotypes and lung disease [5,6,7,8,9], while others did not find any consistent correlation [10,11]. The poor correlation between the severity of the CFTR variants and the severity of the lung disease supports the notion that other cofactors contribute to the modulation of the phenotypic expression of the primary genotype. Due to the direct contact of the lung with microbial pathogens and airborne pollutants, many modulating factors might contribute to the variable lung phenotype observed in patients with the same genotype [12].

An early, sustained, and severe inflammatory process is seen in the airways of pwCF [13], which is characterized by excessive mucus production, chronic bacterial infection, and progressive tissue damage. Airway infection occurs early in the course of the disease; however, there are observations which support that lung inflammation is, at least in part, independent of infections and directly related to defective CFTR [14].

Various CFTR variants related with fatty acid metabolism abnormalities have been reported in CF, such as an abnormally high arachidonic acid (AA) to docosahexaenoic acid (DHA) ratio and a linoleic acid (LA) deficiency, which is directly related to the severity of the CFTR variants [11,15,16,17].

AA released from membrane glycerophospholipids is the rate-limiting step in the enhanced production of eicosanoids such as prostaglandin E_2_ (PGE_2_) [18]. There are two cyclooxygenase (COX) enzymes, COX-1 and COX-2, involved in the conversion of AA to PGE_2_. COX-1 is constitutively expressed in most cells and is involved in the regulation of physiological functions, whereas COX-2 expression is rapidly induced under inflammatory conditions [18]. An increased expression of both COX-1 and COX-2 is found in CF airways [19], which accounts for the increased PGE_2_ production reported in these patients [20,21].

Several findings support the concept that the enhanced COX-2 expression and increased PGE_2_ production found in CF are directly related to CFTR dysfunction rather than to the presence of an inflammatory process associated with chronic bacterial infection [22,23]. Chen et al. [23] demonstrated that the COX-2/PGE_2_ positive feedback loop is negatively regulated by CFTR under normal conditions but augmented with defective CFTR. Borrot et al. showed that CFTR inhibition leads to increased eicosanoid release [24]. Moreover, the absence of CFTR can disrupt cellular signaling networks with broad functional consequences including fatty acid abnormalities [25,26].

Elevated levels of tetranor-PGEM (PGE-M), the PGE_2_ metabolite detected in urine, have been associated with the severity of the CFTR variants [22]. However, marked differences can be found in urine PGE-M levels among patients with similar CFTR variants, suggesting that PGE_2_ production is regulated by factors other than the severity of the mutated receptor [22].

Interestingly, one study found that some *COX-1* and *COX-2* gene polymorphisms were associated with different effects on the severity of lung disease in CF patients with the *F508del* pathogenic variant [27]. However, it is still unclear whether these polymorphisms play any significant role in PGE_2_ production and, thereby, in disease severity.

We hypothesized that the severity of lung disease in CF correlates with the amount of PGE_2_ released, which in turn is related to the severity of the mutated CFTR and further regulated by the presence of some *COX* polymorphisms. We undertook the present study to test this hypothesis.

## 2. Subjects and Methods

### 2.1. Subjects

This study had a cross-sectional design. A total of 30 healthy subjects (12 male, 18 female) aged from 5 to 22 years (11.5 ± 0.75) and 103 pwCF (56 male, 47 female) aged from 4 to 24 years (12.68 ± 0.48) with stable CF were included in this study. PwCF were recruited from a single pediatric CF center (Hospital Universitari Vall d’Hebrón, Barcelona, Spain). The CF diagnosis was established based on clinical data, an abnormal sweat test (sweat chloride > 60 mmol/L), and bi-allelic CFTR pathogenic variants. PwCF were in a stable clinical condition at a regular follow-up visit. Healthy control children were recruited from families of hospital workers. The demographic characteristics of patients and healthy subjects were not statistically different. Clinical and radiological characteristics of pwCF are shown in Table 1. All participants provided informed consent, with parents giving informed consent, prior to enrolment. This study was approved by the institutional Ethics Committee (PR(AMI)143/2013).

### 2.2. Methods

The forced expiratory volume in the first second (FEV_1_) and the forced vital capacity (FVC) were measured by spirometry in pwCF, and the best of three maneuvers, expressed as the percentage of predicted values, was chosen.

Pancreatic sufficiency was defined as the presence of a fecal elastase value > 200 μg/g.

All patients were genotyped using methods reported elsewhere [28]. They were classified into three groups (mild, moderate, and severe) based on the predicted functional consequences of the CFTR protein alteration and on the accepted premise of the dominant phenotypic effect conferred by the milder of the two CFTR pathogenic variants. PwCF carrying Class I, II, and Class III pathogenic variants in their alleles were considered severe, those carrying Class I, II, or III mutations in one allele associated with a Class IV, V, or VI in the second allele were classified as moderate, and those pwCF with any combination of Class IV, V, or VI in both alleles were considered mild. PwCF were divided into three clinical groups according to severity, which was established considering the frequency of upper airway infections, the number of pulmonary exacerbations requiring antibiotic therapy, and the number of pulmonary exacerbations requiring hospitalizations and intravenous antibiotic therapy (Table 1).

Each chest computed tomography (CT) consisted of a volumetric inspiratory and expiratory acquisition. All CTs were scored evaluating the six (lingula as a separate lobe) lung lobes for the presence of central and peripheral bronchiectasis and the extent of trapped air on expiratory CTs. Bronchiectasis was scored as 0 (no bronchiectasis), 1 (one lobe affected), 2 (two lobes affected), or 3 (three or more lobes affected). Similarly, the extension of air trapping was scored from 0 to 3. All scans were scored by a blinded observer radiologist.

Routinely, an airway sample (sputum or cough swab) was collected when pwCF attended the outpatient center every 1–2 months. These samples were incubated in different media for the identification of bacterial and fungal organisms via standard culture protocols.

Urine samples were collected from the participants and stored at −80 °C until analysis. The measurement of PGE-M and PGD-M and final urinary metabolites of PGE_2_ and prostaglandin D_2_ (PGD_2_), respectively, is considered the best method to accurately assess the biosynthesis of PGE_2_ and PGD_2_ generated via the COX pathway [20]. Urinary PGE-M and PGD-M levels were measured using liquid chromatography/tandem mass spectrometry (LC/MS) with slight modifications to the method previously described [21]. Briefly, 1 mL of urine was converted to an O-methyloxime derivative and purified by C18 solid phase extraction before LC/MS analysis. LC was performed on a 2.0 × 50 mm 1.7 µm particle Acquity BEH C18 column (Water Corporation, Barcelona, Spain). Mobile phase A was 95:4.9:0.1 (*v*/*v*/*v*) 5 mM ammonium acetate: [22,23] acetonitrile/acetic acid, and mobile phase B was 10.0:89.9:0.1 (*v*/*v*/*v*) 5 mM ammonium acetate/acetonitrile/acetic acid. The samples were separated by a gradient of 85–76% of mobile phase A over 6 min at a flow rate of 900 μL/min prior to delivery to a 6500 QTRAP (Sciex, Framingham, MA, USA) triple quadrupole mass spectrometer. Urinary creatinine (Cr) levels were measured by a Creatinine Colorimetric Assay kit from Cayman Chemical (Ann Arbor, MI, USA).

The DNA extracted from peripheral blood was analyzed for the presence of different functional variants described in the *COX-1(PTGS1)* and *COX-2(PTGS2)* genes. Both genes were sequenced by NGS using the Generead DNA seq Targeted Panels V2 technique on MiSeq (Illumina, San Diego, CA, USA) equipment. The following prostaglandin polymorphisms were analyzed: *PTGS1* 639 C>A, *PTGS1* 762+14delA, *PTGS2*-899G>C (-765G>C), and *PTGS2* (8473T>C), all the variants were previously reported in both genes [27].

### 2.3. Statistical Analysis

Parametrical statistical methods were used with variables that satisfied the assumption for parametric statistical testing. With variables which did not satisfy this assumption, non-parametric statistical methods were applied to all data sets. For independent samples, comparisons between two groups were carried out using the Mann–Whitney U test, while the Kruskal–Wallis H test was used for multiple groups. The correlation between clinical scale severity and PG values was expressed as Spearman’s rank correlation coefficient. Statistical significance was established at *p* ≤ 0.05.

## 3. Results

The distribution of patients according to *CFTR* gene pathogenic variants’ severity and clinical and radiological characteristics is shown in Table 1. CFTR variants are depicted in Table 2.

### 3.1. Urinary PGE-M and PGD-M Levels

Urine PGE-M and PGD-M concentrations are expressed as medians and interquartile range (25–75th interquartile). Urine PGE-M concentrations were significantly (*p* < 0.0001) higher in pwCF patients (18.10; 7.60–30.50 ng/mg Cr) versus healthy controls (5.65; 3.48–11.48 ng/mg Cr). Similarly, PGD-M levels in pwCF (5.10; 2.50–8.30 ng/mg Cr) were significantly (*p* < 0.01) higher than in healthy controls (2.40; 1.70–5.65 ng/mg Cr).

### 3.2. Correlations between Urinary PGE-M and PGD-M Levels and CFTR Gene Mutation Severity

**Figure 1:**
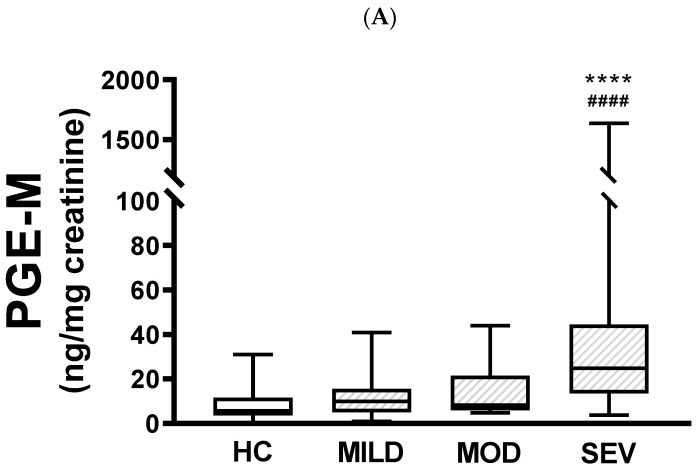

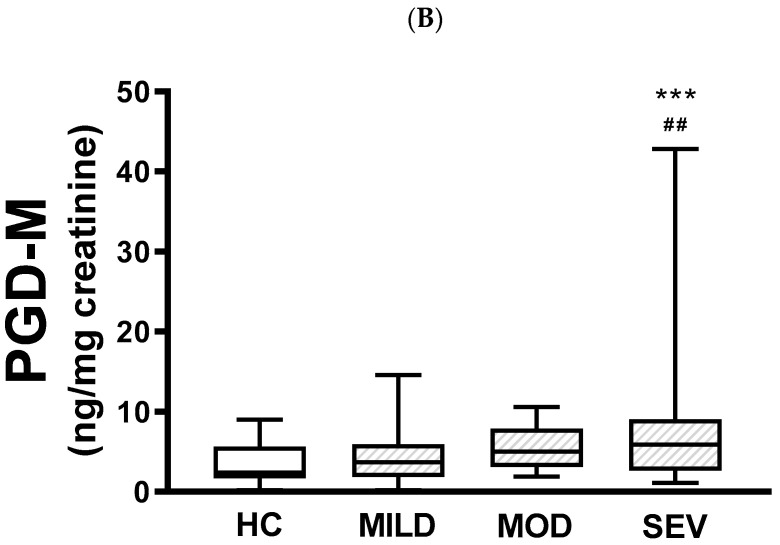
Urinary PGE-M and PGD-M levels and *CFTR* gene pathogenic variants severity. PGE-M (Panel **A**) and PGD-M (Panel **B**) concentrations were analyzed by liquid chromatography/tandem mass spectrometry (LC/MS) in urine samples from healthy controls (HC, N = 30) and patients with mild (MILD, N = 29), moderate (MOD, N = 7), and severe (SEV, N = 65) *CFTR* gene phenotypes. The solid line indicates the median, the box indicates 25–75th percentiles, and whiskers represent the minimum and maximum values. An unpaired *t*-test was used for statistical comparison. **** *p* ≤ 0.0001 and *** *p* ≤ 0.001 compared with HC; #### *p* ≤ 0.0001 and ## *p* ≤ 0.01 compared with MILD.

When urinary PGE-M levels were compared between healthy controls and pwCF with mild, moderate, or severe phenotypes, there were no differences between healthy controls and patients carrying either the mild or moderate phenotype (Figure 1A). In contrast, there were statistically significant differences between the severe phenotype and both healthy controls (*p* < 0.0001) and mild pathogenic variants (*p* < 0.0001). There were no statistically significant differences between mild and moderate phenotypes or moderate and severe phenotypes. Similar results were produced when urinary PGD-M levels were compared, with significant differences only between the severe phenotype and healthy controls (*p* < 0.001) and patients carrying the mild phenotype (*p* < 0.01) (Figure 1B).

### 3.3. Associations between Urinary PGE-M and PGD-M Levels and CF Severity Parameters

In patients with pancreatic insufficiency (N = 70), PGE-M levels (24.25; 12.48–44.50 ng/mg Cr) were higher than in patients with conserved pancreatic function (N = 33) (8.60; 4.60–16.80 ng/mg Cr, *p* < 0.0001). Similarly, PGD-M levels (6.15; 3.00–9.02 ng/mg Cr) were higher than in patients with conserved pancreatic function (3.60; 1.75–5.95 ng/mg Cr, *p* < 0.01).

**Figure 2:**
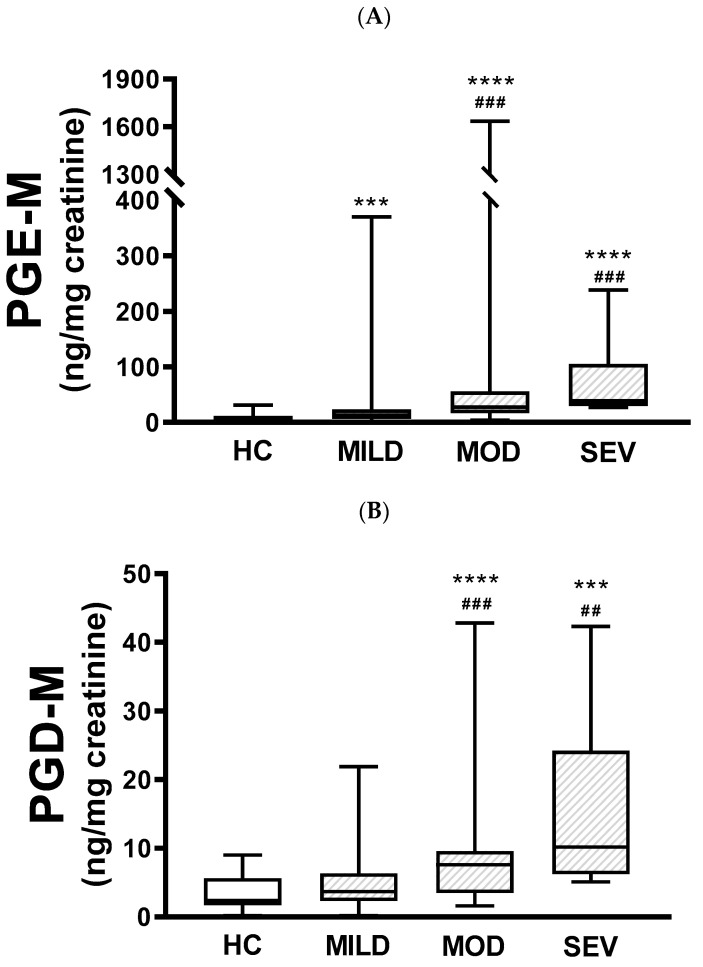
Urinary PGE-M and PGD-M levels and clinical severity. PGE-M (Panel **A**) and PGD-M (Panel **B**) concentrations were analyzed by liquid chromatography/tandem mass spectrometry (LC/MS) in urine samples from healthy controls (HC, N = 30) and patients with mild (MILD, N = 29), moderate (MOD, N = 7), and severe (SEV, N = 65) clinical phenotypes. The solid line indicates the median, the box indicates 25–75th percentiles, and whiskers represent the minimum and maximum values. An unpaired *t*-test was used for statistical comparison. **** *p* ≤ 0.0001 and *** *p* ≤ 0.001 compared with HC; ### *p* ≤ 0.001 and ## *p* ≤ 0.01 compared with MILD.

When urinary PGE-M levels were compared between healthy subjects and pwCF with mild, moderate, or severe clinical severity, there were differences between healthy subjects and pwCF with mild (*p* < 0.001), moderate (*p* < 0.0001), and severe disease (*p* < 0.0001). Also, there were statistically significant differences between mild and moderate disease (*p* < 0.001) and mild and severe disease (*p* < 0.001); however, the difference between moderate and severe was not statistically significant (Figure 2A). Similar results were obtained with PGD-M levels, except that there were no statistical differences between healthy controls and pwCF with mild disease nor between moderate and severe disease (Figure 2B).

There was no correlation between PGE-M levels with either the FEV_1_ (r = −0.1235) or the FVC (r = −0.0964). Similarly, PGD-M levels did not correlate with either the FEV_1_ (r = −0.1901) or the FVC (r = −0.1732).

Since the number of pwCF scoring 2 and 3 was low, the analysis of the presence of bronchiectasis and air trapping was performed binarily (with or without). Patients with bronchiectasis (n = 45) had higher PGE-M (27.80; 16.00–56.30 ng/mg Cr, *p* < 0.0001) and PGD-M (7.00; 3.70–9.90 ng/mg Cr, *p* < 0.001) levels than patients without bronchiectasis (n = 57) (PGE-M: 12.70; 6.45–20.35 ng/mg Cr; PGD-M: 3.70; 2.30–6.45 ng/mg Cr).

The presence of air trapping in the CT scan (n = 64) was associated with higher PGE-M levels (22.25; 11.80–41.95 ng/mg Cr) than in those without radiological findings of air trapping (n = 28) (11.65; 6.37–27.68 ng/mg Cr, *p* < 0.05). In contrast, there were no differences in PGD-M levels between patients with and without air trapping.

### 3.4. Associations between Urinary PGE-M and PGD-M Levels, Airway Infections, and a Docosahexaenoic (DHA)-Supplemented Diet

PwCF with airways chronically colonized by fungi (n = 15) had similar urinary PGE-M and PGD-M levels as non-colonized patients (n = 87).

Patients with a DHA-supplemented diet (n = 5) had lower urinary PGE-M levels (6.60; 4.90–14.70 ng/mg Cr) than those without a supplemented diet (n = 96) (19.10; 7.80–32.95), but the difference was not statistically significant (*p* = 0.058). On the other hand, there were no differences in urinary PGD-M levels between the two groups.

### 3.5. Associations between Prostaglandin Polymorphisms with CF Severity and Urinary Prostaglandin Levels

The four polymorphisms were analyzed in 102 pwCF. The *COX1*-639 C>A polymorphism was identified as homozygous in 4 patients (3.9%), heterozygous in 17 (16.7%), and negative in 81 (79.4%).

The *COX1*-762+14delA polymorphism was identified as homozygous in 1 pwCF (1%), heterozygous in 17 (16.7%), and negative in 84 (82.4%).

The *COX-2*-765G>C polymorphism was identified as homozygous in 4 pwCF (3.9%), heterozygous in 31 (30.4%), and negative in 67 (65.7%).

The *COX-2*-8473T>C polymorphism was identified as homozygous in 9 pwCF (8.8%), heterozygous in 40 (39.2%), and negative in 53 (52%).

When comparing the four polymorphisms with the different variables: clinical severity, genetic variants, pancreatic function, lung function, and the presence of air trapping and/or bronchiectasis with the chest CT, no significant differences were. In addition, there were no significant differences between the presence of polymorphisms and chronic colonization by *Staphyloccocus aureus* (SA), *Pseudomonas aeruginosa* (PA), and PGE-M and PGD-M urinary levels.

## 4. Discussion

Forty years ago, Charlotte M. Anderson hypothesized that a disturbed PGE_2_ metabolism could be involved in CF [29]. Supporting this hypothesis, several studies have reported the ability of CFTR to regulate COX-2 expression and, thereby, PGE_2_ biosynthesis [19,20,21,22,23]. A defective CFTR protein leads to enhanced COX-2 expression resulting in an increased release of PGE_2_ in CF patients [19,21,23]. Moreover, it was shown that CFTR inhibition leads to membrane destabilization, favoring eicosanoid synthesis [24]. In addition to its role as an ion channel, CFTR also forms complexes with a host of signaling proteins (kinases and phosphatases) involved in fatty acid metabolism [25,26].

Given the direct relationship reported between CFTR dysfunction and increased PG synthesis, we hypothesized that the amount of PG released in CF patients would be a marker of the severity of CFTR dysfunction. According to this hypothesis, PG production should associate with parameters of CF severity.

Our study resulted in several main findings: (a) there is a limited relationship between the severity of CFTR genetic dysfunction and PG production; (b) exocrine pancreatic insufficiency is closely associated with the severity of CFTR dysfunction and PG production; (c) there is no correlation between PG levels and lung function parameters; (d) PG production associates with both clinical status and radiological findings (bronchiectasis and air trapping); and (e) *COX-1* and *COX-2* gene polymorphisms do not appear to contribute to the regulation of PG synthesis.

Previous studies found moderate or no correlation between CFTR severity and PG production. Moreover, there are notable differences between urinary PG levels in patients with the same pathogenic variants, which suggest that factors other than mutation severity are involved in the regulation of PG metabolism. These factors remain to be elucidated. A previous study suggested the potential role of *COX-1* and *COX-2* gene polymorphisms in the clinical severity of pwCF harboring the *F508del* mutation [23]; however, we found no relationship between *COX* polymorphisms and either severity parameters or urinary levels of PGE-M and PGD-M that reflect systemic prostanoid production.

In keeping with previous reports, our study shows a relationship between the presence of normal or defective exocrine pancreatic function and the severity of CFTR pathogenic variants and urinary PG levels [20].

FEV_1_ measurement has been a central outcome measurement for clinical management and trials. However, various studies concluded that the FEV_1_ has limited sensitivity in detecting disease severity and monitoring disease progression [30,31]. Our study replicates previous observations regarding the lack of correlation between lung function parameters (FEV_1_ and FVC) and PG biosynthesis in CF patients [21]. Based on the concept that PG biosynthesis is associated with the severity of *CFTR* dysfunction, our results lend further support to studies reporting that lung function assessment has a limited value in the evaluation of CF severity.

In our study, we found that urinary PGE-M levels were associated with the clinical severity of the disease, detecting significant differences between healthy controls and pwCF with mild, moderate, and severe clinical severity. We also found significant differences between mild and moderate CF disease and mild and severe CF disease. Various recent studies support that bronchiectasis detected by chest CT is a sensitive indicator of prognosis, pulmonary exacerbations, and mortality in pwCF patients [32,33,34]. Compared with bronchiectasis, the potential relevance of air trapping as a marker of disease severity remains to be clearly established. Nevertheless, a previous study validated the presence of trapped air in the CT scan as an independent predictor of pulmonary exacerbations [35]. We found marked differences in PG levels between pwCF with and without bronchiectasis in the CT scan. Moreover, the presence of air trapping was also associated with higher levels of PGE-M than in those without radiological findings of air trapping. Taken together, clinical and radiological findings support the hypothesis that the level of PG production is a potential marker of disease severity.

Interestingly, and in accordance with our results, a very recent study found that PGE_2_ levels in bronchoalveolar lavage fluid collected from the lung of CF patients correlated positively and significantly with disease progression; the higher the PGE_2_ levels, the faster the progression of the disease [36].

Chronic infection can stimulate *COX-2* expression and, therefore, induce PG release. However, in our study, we did not find any difference in urinary PG levels between chronically colonized and non-colonized patients.

A high-dose DHA supplementation diet can improve clinical outcomes, such as disease exacerbations associated with a decrease in inflammatory markers [25]. In our study, we found reduced urinary PG levels in pwCF on a supplemented DHA diet compared with those not on this diet, but the difference only tended towards statistical significance possibly because only a few patients from our cohort were on this therapy. In a very recent study, the effects of a supplemented DHA diet on urinary PGE_2_ levels were assessed and compared with a control placebo diet. There were no differences in the impact of the supplemented diet and placebo on PGE_2_ production. The study, however, was carried out in a small sample of pwCF with low baseline urinary PGE_2_ levels. Thus, a more extensive clinical trial will be required to assess the effects of this therapy on PG production.

A recent study investigated the effects of ivacaftor on urinary PGE-M levels in pwCF with the *G551D* mutation. Ivacaftor treatment significantly decreased the urine levels of PGE-M, suggesting urinary PGE-M as an interesting marker to assess the efficacy of new CF therapies [37].

The mechanisms underlying the relationship between defective CFTR function and excessive PGE_2_ synthesis remain to be fully elucidated. We are tempted to speculate that the increased PGE_2_ release in CF is intended to stimulate the activity of defective CFTR. When CFTR recovers its function, at least partially, with some of the new therapies, the excess PGE_2_ production is no longer needed.

Our study has some limitations such as not having considered the symptoms of other organs or systems affected in pwCF such as the gastrointestinal tract. Similarly, sweat chloride levels were not taken into account, a test that is related to the severity of the disease and that has been shown to be useful to assess the effectiveness of new therapies with CFTR modulators.

In summary, there is a significant need to develop new, relevant biomarkers to monitor clinical care prognosis and evolution as well as the effects of new therapies in CF. Taken together, the data reported in the present study suggest that measuring urinary PG levels could fulfill this role. On the other hand, we found no relationship between *COX* polymorphisms with either severity parameters or urinary levels of PGE-M and PGD-M that reflect systemic prostanoid production.

## Figures and Tables

**Table 1:** Clinical and radiological characteristics of cystic fibrosis patients.

FVC, % pred (n = 93)	104.1 ± 1.8
FEV_1_, % pred (n = 93)	94.3 ± 2.3
Pancreatic insufficiency, (n = 103) %	70 (68%)
Severity of *CFTR* gene mutation (n = 101):	
Mild, %	28.7%
Moderate, %	6.9%
Severe, %	64.4%
Clinical severity (n = 101):	
Mild, %	64.4%
Moderate, %	29.7%
Severe, %	5.9%
Bronchiectasis, (n = 102) %	45 (44%)
Air Trapping (n = 92) %	64 (69.%)

Data presented as mean ± SEM or percentage. FVC, forced vital capacity; pred, predicted; FEV_1_, forced expiratory volume in 1 s; CFTR, cystic fibrosis transmembrane conductance regulator.

**Table 2:** Distribution of *CFTR* gene variants (N = 103).

F508del homozygote, n (%)	19 (18.4%)
F508del heterozygote, n (%)	59 (57.3%)
Other pathogenic variants, n (%)	25 (24.3%)

## Data Availability

Data is unavailable due to ethical restrictions.
